# Heparin is required for the formation of granules in connective tissue mast cells

**DOI:** 10.3389/fimmu.2022.1000405

**Published:** 2022-11-09

**Authors:** Sandra Abril Herrera-Heredia, Hsuan-Po Hsu, Cheng-Yen Kao, Yu-Huan Tsai, Yu Yamaguchi, Axel Roers, Chia-Lin Hsu, Ivan L. Dzhagalov

**Affiliations:** ^1^ Institute of Microbiology and Immunology, National Yang Ming Chiao Tung University, Taipei, Taiwan; ^2^ Sanford Burnham Prebys Medical Discovery Institute, La Jolla, CA, United States; ^3^ Institute for Immunology, Heidelberg University Hospital, Heidelberg, Germany

**Keywords:** Heparin, mast cells, granules, *Mrgprb2*, irritant dermatitis, necrotizing dermatitis

## Abstract

Mast cells are innate immune cells strategically positioned around blood vessels near body surfaces. Their primary weapons are bioactive amines, mast cell-specific proteases, and cytokines stored in preformed granules. Mast cells granules constituents are packaged efficiently with the help of the highly negatively charged Heparan sulfate-derivative, Heparin. Heparin is one of the most widely used drugs to treat coagulation disorders, yet, it is not found in the circulation at a steady state, casting doubt that the prevention of blood clotting is its physiological function. Early studies using *Ndst2* ^-/-^ mice have shown that Heparin is essential for mast cells granules formation. However, these mice could still produce less sulfated Heparan sulfate that could potentially replace Heparin. Here, we have created and validated a novel genetic model for Heparin deficiency, specifically in connective tissue mast cells, to address the physiological role of this molecule. Using this model, we have demonstrated that Heparin is required for mast cell granules formation; without it, mast cells are reduced in the peritoneal cavity and the skin. The absence of Heparin impaired the response to passive cutaneous anaphylaxis but, surprisingly, enhanced ear swelling in an irritant dermatitis model and reduced the lesion size and bacterial burden in a *Staphylococcus aureus* necrotizing dermatitis model. The altered function of Heparin-deficient mast cells in the latter two models was not mediated through enhanced Histamine or TNFα release. However, the *Mrgprb2* receptor was up-regulated in knock-out mast cells, potentially explaining the enhanced response of mutant mice to irritant and necrotizing dermatitis. Altogether our results expand our current understanding of the physiological role of Heparin and provide unique tools to further dissect its importance.

## Introduction

Mast cells (MCs) are tissue-resident innate immune cells that play essential roles in allergic reactions and toxin neutralization (1). In rodents, they can be divided into two subsets: connective tissue MCs and mucosal MCs (2). Connective tissue MCs are present in the skin, peritoneal cavity, and most connective tissues and produce the glycosaminoglycan Heparin and a specific set of MC proteases. Mucosal MCs are positioned at the mucosal surfaces of the intestine and the lungs, contain Chondroitin sulfate, and produce a distinct set of proteases. Upon activation, MCs can quickly release preformed mediators that can induce immediate and powerful effects at local and systemic levels that can kill humans and animals within minutes – a phenomenon called anaphylaxis, which is MCs most well-known function. The search for positive effects of MCs led to the discovery that MCs do not develop in c-kit mutant mice, which has resulted in many studies showing wide-ranging activities of MCs (3). However, many of these effects have been questioned because c-kit is essential for the development and function of other cell types as well (4). In recent years, the field of MC biology has been undergoing a re-evaluation of their functions using c-kit-independent models (5, 6).

MCs’ primary weapons are their granules that contain many mediators (7). These include bioactive amines, of which Histamine is the most important; several mast cell-specific proteases such as Mcpt1, Mcpt4, Mcpt5, Mcpt6, and Cpa3; and cytokines such as TNFα, IL-4, VEGF, Fgf2. These MCs granules constituents are all positively charged and are packaged together with the help of Heparin – a highly negatively charged Heparan sulfate (HS) derivative (8). Heparin is produced only in MCs and is an important drug used to treat coagulation disorders (9). It activates Serpin C1 (Anti-Thrombin III) and limits blood clotting. However, no Heparin could be found in the blood of people or animals, and it appears that its physiological function is not related to coagulation.

The first glimpse into the physiological role of Heparin came from two studies that deleted the enzyme N-deacetylase/N-sulfotransferase 2 (*Ndst2*), which is essential for the sulfation of HS to produce Heparin (10, 11). The authors found that in the absence of Heparin, the granules of MCs were largely missing, and some of the mediators stored in the granules were greatly reduced. However, the two studies differed in some important aspects. Forsberg et al. found that the MCs were substantially reduced in the peritoneal cavity of *Ndst2* ^-/-^ mice, and they contained significantly less Histamine than controls (10). In contrast, Humphries et al. reported unchanged numbers of MCs and Histamine content (11). One limitation of both studies is that although Heparin could not be produced in the mutant animals, they could still make HS with a lesser degree of sulfation that could potentially substitute for Heparin in MCs.

The physiological importance of Heparin has also been studied in mice lacking Serglycin – a protein rich in Serine and Glycine that is the physical carrier of Heparin chains in MCs. Serglycin knock-out mice were found to have fewer granules, and many granule constituents such as MC-specific proteases, Histamine, and Serotonin were significantly reduced (12, 13). However, using the same animal model, Abrink et al. reported that the peritoneal MCs were almost absent in *Serglycin* ^-/-^ mice (12), while Ringvall et al. found a typical abundance of peritoneal MCs (13). These mice also had decreased clearance of *Klebsiella pneumoniae* infection (14) but a normal response to Lymphocytic choriomeningitis virus infection (15). However, Serglycin is expressed in many hematopoietic and non-hematopoietic cells, and the phenotype in the knock-out mice cannot be attributed solely to the lack of Heparin in MCs (16). Moreover, Serglycin can carry other glycosaminoglycans in addition to Heparin, such as Chondroitin sulfate. Thus, its deletion might impair functions that are not dependent on Heparin.

Altogether, although the studies of *Ndst2* ^-/-^ and *Serglycin* ^-/-^ mice have provided valuable insight into what does Heparin do in our body, the discrepancies between studies using the same animal model and the limitations of different genetically deficient mouse strains leave open the question: What is the physiological function of Heparin? Here, we addressed the role of Heparin by crossing *Mcpt5* ^Cre^ mice with *Ext1* ^f/f^ and, thus, abolishing HS and Heparin synthesis, specifically in connective tissue MCs. Heparin-deficient MCs had significantly reduced granule content, including Histamine and TNFα, and their numbers in the peritoneal cavity and the skin were diminished considerably. Interestingly, although the reduced number of MCs and their granules resulted in weaker passive cutaneous anaphylaxis (PCA), the response of mutant mice to skin irritants and *S. aureus* necrotizing dermatitis was enhanced in part due to the up-regulation of the *Mrgprb2* receptor on MCs. Taken together, our studies provide important insights into the physiological functions of Heparin and the adaptations of MCs to its absence.

## Material and methods

### Mice

C57BL/6 mice were purchased from the National Laboratory Animal Center (Taipei, Taiwan). *ROSA26* ^LSL-GFP^ (B6.Cg-Gt(ROSA)26Sor^tm6(CAG-ZsGreen1)Hze^/J, JAX stock# 007906) mice were purchased from The Jackson Laboratory (17). *Ext1* ^f/f^ (C57BL/6-Ext1^tm1Yama^) (18) and *Mcpt5* ^Cre^ (Tg(Cma1-cre)ARoer) mice have been previously described (19). All mice were housed in the animal facility of NYCU and used between 6 and 14 weeks of age. All animal experiments were approved by the Institutional Animal Care and Use Committee (IACUC) of NYCU.

### Cell isolation

Peritoneal cells were obtained by injecting 3 mL of cold phosphate-buffered saline (PBS) followed by a gentle massage of the peritoneal cavity. Cells were extracted using a syringe, washed, and resuspended in complete RPMI (cRPMI) medium containing 10% Fetal Bovine Serum, 1% Penicillin/Streptomycin, 1% L-Glutamine, and 0.1% 2-Mercaptoethanol (all from Gibco). For skin cell isolation, ears were cut, and dorsal and ventral sheets were separated and finely minced with scissors. The tissue pieces were collected and digested in cRPMI medium containing 1.0 mg/mL Collagenase P (Roche) and 0.1 mg/mL DNase I (Sigma) at 37°C for 40 min in constant agitation. The cells were filtered through 70 μm Nylon mesh (Small Parts) and washed with PBS. Cells were resuspended in 500 μL of 1X ACK (Ammonium-Chloride-Potassium) lysis buffer, incubated for 45 sec at room temperature (RT), washed, and resuspended in cRPMI. Small intestines were harvested, flushed with PBS, opened, and cut into 1 cm long pieces. Tissue pieces were transferred into a 50 mL conical tube and incubated in HBSS without Ca^2+^ and Mg^2+^ (Gibco) supplemented with 5 mM EDTA (Merck), 10 mM HEPES (Goldbio), and 5% Fetal Bovine Serum (Gibco) for 20 min at 37°C in constant agitation. Tissue pieces were washed with PBS and digested in cRPMI containing 1.0 mg/mL Collagenase P (Roche) and 0.1 mg/mL DNase I (Sigma) at 37°C for 40 min in constant agitation. Cells were passed through 100 and 70 μm cell strainers (Biologix) and washed twice, resuspended in 2 mL of 1X ACK buffer, incubated for 1 min at RT, washed and resuspended in cRPMI.

### Flow cytometry

Single-cell suspensions were blocked with 100 μL supernatant from 2.4G2 hybridoma (a kind gift by Dr Fang Liao, Academia Sinica, Taipei, Taiwan) and stained with antibodies for 20 min on ice in 100 μL FACS buffer [PBS + 0.5% Bovine Serum Albumin (BSA, Bionovas) + 1 mM EDTA (Merck) + 0.1% NaN3 (Sigma)]. The following antibodies were used: anti-mouse CD45 PerCP/Cy5.5 (clone 30-F11), anti-mouse CD11b BV785 (clone M1/70), anti-mouse CD11b FITC (clone M1/70), anti-mouse TCRβ APC (clone H57-597), anti-mouse CD117 BV421 (clone 2B8), anti-mouse CD117 Pe/Cy7 (clone ACK2), anti-mouse CD5 Pe/Cy7 (clone 53-7.3), anti-mouse MHCII APC/Fire 750 (clone M5/114.15.2), anti-mouse FcεR1α PerCP/Cy5.5 (clone MAR-1), anti-mouse FcεR1α APC (clone MAR-1), anti-mouse B220 Biotin (clone RA3-6B2), anti-mouse B220 BV711 (clone RA3-6B2), anti-mouse Gr-1 APC/Cy7 (clone RB6-8C5), anti-mouse F4/80 BV421 (clone BM8), anti-mouse ICAM2 FITC (clone MIC2/4), anti-mouse Ly6c BV711 (clone HK1.4), anti-mouse Ly6c BV700 (clone HK1.4), anti-mouse Ly6c Biotin (clone HK1.4), anti-mouse CD3ε APC/Fire 750 (clone 145-2C11), anti-mouse CD3ε biotin (clone 145-2C11) (all from BioLegend). After staining, cells were washed with FACS buffer and, if required, incubated with Streptavidin PE or Streptavidin APC/Cy7 (BioLegend). The cells were washed and resuspended in FACS buffer containing Propidium Iodine (Sigma) or DAPI (ThermoFisher). To detect Heparin by Serpin C1 binding, cells were incubated with Zombie Aqua (BioLegend) for 30 min, washed, and stained with the required antibodies for surface markers. Later, cells were fixed with 2% paraformaldehyde (PFA, Electron Microscopy Sciences) for 15 min at RT and washed with FACS buffer. For permeabilization, cells were incubated in 500 μL of permeabilization buffer [PBS + 1% Triton X-100 (Sigma)], washed, incubated for 30 min with biotinylated Serpin C1, washed, and incubated with Streptavidin PE or Streptavidin APC (BioLegend) as required. Finally, cells were washed and resuspended in FACS buffer. The data were acquired on LSR Fortessa (BD Biosciences) flow cytometer running Diva 8 software and analyzed with FlowJo 10.7.1 (BD Biosciences).

### Serpin C1 biotinylation

Serpin C1 protein (MyBioSource) was diluted in binding buffer [10 mM sodium phosphate (Bionovas) + 150 mM NaCl (Sigma), pH 7.4], and loaded into a pre-equilibrated HiTrap Heparin HP column (GE Healthcare). The flow-through was reloaded into the column twice to ensure complete binding, and then the column was washed with binding buffer. EZ-Link™ Sulfo-NHS-LC-biotin (Thermo Scientific) was prepared and applied in 50-fold molar excess of Serpin C1 per column volume and incubated for 1 hour at RT. The reaction was terminated by washing the column four times with termination buffer [Binding buffer + 100 mM glycine (Sigma), pH 7.4]. Then, the column was washed with binding buffer. The biotinylated Serpin C1 was eluted with elution buffer [10 mM sodium phosphate + 4 M NaCl, pH 7.4]. The whole procedure was performed on ÄKTA (GE Healthcare) protein purification system. Biotinylated Serpin C1 was dialyzed and concentrated in Dulbecco’s Phosphate Buffered Saline (DPBS, Gibco) with a Vivaspin 6 MWCO 3 kDa spin column (Sartorius). Finally, Biotinylated Serpin C1 aliquots were prepared in DPBS containing 2.0% glycerol (MP Biomedicals) and 0.2% BSA (Bionovas) and preserved at –20°C until use.

### Immunofluorescent microscopy

Shaved back skin was dissected and fixed for 2 hours in 4% PFA (Electron Microscopy Sciences)/10% sucrose (Sigma) at 4°C, followed by overnight incubation in 30% sucrose (Sigma) at 4°C. After fixation, the tissue was embedded in Tissue-Tek O.C.T compound (Sakura Fintek), frozen, and stored at -80°C until use. Skin sections were cut with a thickness of 10 μm at -20°C in a cryostat microtome (NX50, Thermo Scientific). The sections were fixed in acetone (Honeywell) for 10 minutes at -20°C, air-dried, and blocked with 5% donkey serum (Jackson ImmunoResearch) in PBS + 0.25% Triton X-100 (Sigma) for 2 hours at room temperature, followed by overnight incubation at 4°C in the dark with rabbit polyclonal anti-Histamine antibody (GeneTex) diluted in 2.4G2 supernatant. After incubation, sections were washed 3 times and incubated for 2 hours at room temperature in the dark with AF555 donkey anti-rabbit antibody (Invitrogen) diluted in 2.4G2 supernatant, washed, stained with DAPI (Thermo Fisher) for 5 minutes at RT, washed, and mounted in glycerol (Sigma) containing 0.1% n-propyl gallate (Sigma). Images were obtained with the Axio Observer 7 microscope (Zeiss) equipped with ApoTome.2 and Axiocam 702 monochrome camera (both from Zeiss). Image analysis was performed using the “Spot” function of Imaris 8.0.2 (Bitplane).

### Avidin staining

Avidin was stained as previously described (20). Briefly, skin sections were fixed in acetone (Honeywell) for 10 min at -20°C and air-dried. Avidin conjugated with AF488 (Invitrogen) was diluted in immunohistochemistry buffer [0.1 M Tris (Bionovas) + 0.5% w/v BSA (Bionovas), pH 7.4] containing 2% Triton X-100 (Sigma) and 0.5% skim milk. Sections were incubated with Avidin-AF488 overnight at RT in the dark with constant agitation. Then the sections were washed with immunohistochemistry buffer and stained with DAPI (Thermo Fisher) for 5 min at RT washed and mounted in glycerol + 0.1% n-propyl gallate.

### Cytospin and histological stainings

Ear and peritoneal cavity single-cell suspensions were enriched with CD117 conjugated magnetic beads (Miltenyi) and mounted with a cytospin (Cytospin 3, Shandon) onto Superfrost Plus slides (Thermo Scientific) under the following conditions: 500 rpm for 5 min. The slides were air-dried and stained with Giemsa (Merck) or Toluidine Blue O (Sigma) following standard procedures. Images were obtained with the Axio Observer 7 microscope equipped with Axiocam 702 monochrome camera (Zeiss).

### Bone marrow-derived mast cells

Bones were dissected aseptically from 14-weeks-old mice, and the marrow was flushed in complete Iscove’s modified Dulbecco (cIMDM) medium supplemented with 10% Fetal Bovine Serum, 1% Penicillin/Streptomycin, 1% L-Glutamine, and 0.1% 2-Mercaptoethanol (all from Gibco). The cells were cultured in cIMDM media for at least 4 weeks at 37°C in 5% CO_2_ with 10 ng/mL of recombinant murine IL-3 (PreproTech) or until the purity of MCs (defined as CD117^+^ and FcεR1^+^) reached > 90%. Purity was assessed weekly by flow cytometry.

### *Ext1* copy number

Peritoneal cavity single-cell suspensions were enriched with CD117 conjugated magnetic beads (Miltenyi) and sorted on BD FACSMelody (BD Biosciences) cell sorter running BD FACSChorus (BD Biosciences) software version 1.3.3 after labeling with antibodies described in the flow cytometry section. Sorted cells were incubated with proteinase K (Arrowtec) at 60°C for 5 min to lyse the cells, followed by the extraction of the genomic DNA using the gSYNC extraction kit (Geneaid) following the manufacturer’s instructions. *Ext1* and *Tfrc* genes were amplified together using TaqMan Copy Number Assay ID Mm00613274_cn labeled with FAM and TaqMan Copy Number Reference Assay (cat. # 4458366 labeled with VIC), respectively (both from ThermoFisher) in a PCR thermal cycler (StepOne Real-Time, Applied Biosystems) with the following conditions: 1 cycle of denaturation/enzyme activation at 95°C for 10 min, 40 cycles of denaturation at 95°C for 15 sec followed by annealing/extension at 60°C for 1 min. *Ext1* and *Tfrc* genes expression were analyzed on StepOne (Applied Biosystems) software version 2.3, and fold increase was calculated using the comparative CT (ΔΔCT) method on Microsoft Excel (Microsoft). *Ext1* expression in each sample was normalized using the *Tfrc* gene expression.

### *Mrgprb2* expression

Skin single-cell suspensions were stained as described in the flow cytometry section and sorted with FACSMelody cell sorter (BD Biosciences) running BD FACSChorus (BD Biosciences) software version 1.3.3. CD45^+^CD3^-^CD11b^-^FcεR1^+^c-kit^+^ sorted cells were directly collected in TRI Reagent (Invitrogen) for RNA extraction. Cells were lysed and homogenized in TRI reagent (Invitrogen) by pipetting and incubated for 5 min at RT. 10% of 1-bromo-3-chloropropane (BCP, Sigma) was added, samples were mixed, incubated for 10 min at RT, and centrifuged. The aqueous phase was transferred to a new tube, and RNA was precipitated with isopropanol (Sigma). 1 μg/μL of glycogen (Gene M) was added as an RNA carrier, and samples were incubated overnight at -80°C. Pellets were washed twice with 75% ethanol (J.T. Baker), air-dried, and resuspended in nuclease-free water (Invitrogen). cDNA was synthesized from the isolated RNA using the High-Capacity RNA to cDNA kit (Applied Biosystems) in a thermal cycler (C-1000 Touch, Biorad) with the following conditions: 1 cycle at 37°C for 60 min and 1 cycle at 95°C for 5 min. *Mrgprb2* and *Rpl19* mRNA were amplified together using TaqMan Gene Expression Assays: for *Mrgprb2* Assay ID: Mm01701139_m1 labeled with FAM and for *Rpl19* – Assay ID: Mm02601633_g1 labeled with VIC, primer-limited (both from ThermoFisher)] in a PCR thermal cycler (StepOne Real-Time, Applied Biosystems) with the following conditions: 1 Hold cycle at 50°C for 2 min, 1 Denaturation cycle at 95°C for 10 min and 40 cycles of denaturation at 95°C for 15 sec and annealing/extension 60°C for 1 min, respectively. *Mrgprb2* and *Rpl19* genes expression were analyzed on StepOne (Applied Biosystems) software version 2.3, and fold increase was calculated using the comparative CT (ΔΔCT) method on Microsoft Excel (Microsoft). The expression of *Mrgprb2* in each sample was normalized using the *Rpl19* gene expression.

### Sterile peritonitis model

Mice were injected intraperitoneally with 1 mL of 4% Brewer modified Thioglycolate medium (Sigma). Four days post-injection, the peritoneal cells were obtained as described in the Cell Isolation paragraph and analyzed by flow cytometry.

### Bacterial strains

*Listeria monocytogenes* expressing chicken Ovalbumin was purchased from Sungyee Biotechnology (Nanjing, China) (21). The bacteria were cultured overnight at 37°C in constant agitation in Brain Heart Infusion (BHI, BD Diagnostic Systems) broth containing 5 μg/mL of erythromycin (Sigma) and then diluted 1:20 in fresh BHI broth (BD Diagnostic Systems) until the culture reached the log phase. Frozen stocks were prepared in BHI broth containing 20% glycerol (MP Biomedicals) and stored at -80°C until use. *Staphylococcus aureus* strain JE2 (BEI Resources, NIH, USA) (22) was cultured in Tryptone Soy Broth (TSB, Neogen) overnight at 37°C under constant agitation and then diluted at 1:100 in fresh TSB (Neogen) until the culture reached the log phase. Stocks were prepared in TSB containing 20% glycerol (MP Biomedicals) and stored at -80°C until use. Prior to mice infection, stocks were thawed on ice, washed, and resuspended in sterile PBS.

### *Listeria monocytogenes* infection model

Mice were injected intraperitoneally with 10^7^ CFUs of *L. monocytogenes* in 100 μL of sterile PBS. On day 2 or 7 post-infection, mice were sacrificed, spleen and liver were harvested and placed in a 14 mL polypropylene round-bottom tube (Falcon) filled with cold, sterile PBS. Organs were homogenized with a high-speed homogenizer system (T25, IKA). To determine bacterial burden, serial dilutions of the homogenates were plated on BHI agar (BD Biosciences and Amresco) plates, incubated overnight at 37°C, and colonies were manually counted.

### *Staphylococcus aureus* infection model

Two–three days before infection, mice were anesthetized with ketamine (Toronto Research Chemicals) and xylazine (Sigma), backs were shaved with a clipper (MB-021, Urbaner), and hair was removed with a hair removal cream (Nair). For infection, mice were injected intradermally with 10^7^ CFUs of *S. aureus* in 30 μL of sterile PBS. Lesions were measured every two days starting on day 1 and up to day 9 as follows: Lesions borders were manually marked on tracing paper, and the total area was calculated using ImageJ after digital scanning of the tracers’ sheets. To determine the bacterial burden, mice were sacrificed on day 3 and 10 post-infection, 2 – 3 mm of the skin surrounding the lesions was harvested, weighed, and placed in a 14 mL polypropylene round-bottom tube (Falcon) with cold, sterile PBS. Harvested skin was homogenized using a high-speed homogenizer system (T25, IKA). Serial dilutions of the homogenate were plated in Tryptic Soy Agar (BD Diagnostic Systems and Amresco) plates, incubated overnight at 37°C, and colonies were manually counted. The number of colonies was normalized to the weight of the tissue for each sample.

### Irritant cutaneous dermatitis

Mice were treated with 50 μL of 0.5% of 1-Fluoro-2,4-dinitrobenzene (DNFB, Sigma) in acetone/olive oil (4:1) (25 μL each side of the ear). Ear thickness was measured with an analog micrometer (Mitutoyo 103-129).

### Passive cutaneous anaphylaxis

Mice ears were injected intradermally with 100 ng of αOVA-IgE antibody (clone E-C1, Chondrex, Inc). The following day mice were injected intravenously with 100 μg of OVA (Sigma). Ear thickness was measured with an analog micrometer (Mitutoyo 103-129).

### Statistical analysis

Data analyses were performed in Prism 9 (GraphPad). All data were analyzed with an unpaired Student t-test except for the bacterial burden in **Figure 4F** and **Figures 5C, D**, which was analyzed with a non-parametric Mann-Whitney test, and presented as mean ± SEM. Statistical significance was defined as follows: *p < 0.05, **p <0.01; ***p <0.001 and ****p < 0.0001. Graphs were created with Prism 9 (GraphPad) and Adobe Illustrator 2022 (Adobe).

## Results

### Connective tissue but not mucosal MCs express *Mcpt5* ^Cre^

**Figure 1:**
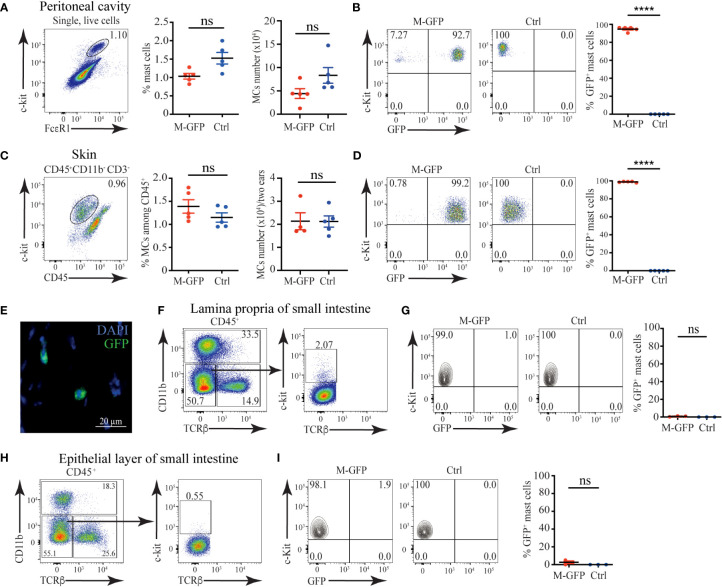
Connective tissue MCs are efficiently labeled in *Mcpt5^Cre^* X *ROSA26^LSL-GFP^* (M-GFP) mice. **(A)** Representative gating strategy, percentages, and numbers of c-kit^+^FcεR1^+^ MCs among peritoneal exudate cells in M-GFP and control mice determined by flow cytometry. **(B)** Representative flow cytometry plots and percentages of GFP^+^ peritoneal MCs in M-GFP and control mice. **(C)** Representative gating strategy, percentages, and numbers of skin MCs in M-GFP and control mice determined by flow cytometry. **(D)** Representative flow cytometry plots and percentages of GFP^+^ skin MCs in M-GFP and control mice. **(E)** Representative immunofluorescent microscopy image of skin MCs in M-GFP mice. **(F)** Representative gating strategy and percentages of MCs among CD45^+^ cells in lamina propria of the small intestine of M-GFP and control mice. **(G)** Representative flow cytometry plots and percentages of GFP^+^ MCs in lamina propria of the small intestine of M-GFP and control mice. **(H)** Representative gating strategy and percentages of MCs among CD45^+^ cells in the epithelial layer of the small intestine of M-GFP and control mice. **(I)** Representative flow cytometry plots and percentages of GFP^+^ MCs in the epithelial layer of the small intestine of M-GFP and control mice. Results are represented as mean ± SEM. Each symbol in the graph represents an individual mouse. Flow cytometry plots in **(A–D)** are representative of 5 individual experiments, while the plots in **(F–H)**, and **(I)** are representative of 3 individual experiments. The image in **(E)** is representative of 3 different mice. Statistical significance was determined by unpaired Student’s t-test. Values of p < 0.05 were considered statistically significant, ****p < 0.0001, ns, not significant.

To ablate all Heparan sulfate and Heparin specifically in MCs, we decided to use *Mcpt5* ^Cre^ mice (19). As a first step, we tested the specificity of this model by crossing *Mcpt5* ^Cre^ to *ROSA*26^LSL-GFP^ mice (17) and examining the degree of labeling of MCs with GFP. Among peritoneal lavage cells, the c-kit^+^FcεR1^+^ MCs were ~1.5% of all cells, and they numbered ~8x10^4^ (**Figure 1A**). The presence of the two transgenes in *Mcpt5* ^Cre^ X *ROSA26* ^LSL-GFP^ (M-GFP) mice did not significantly change MCs proportions and numbers, but more than 90% of peritoneal MCs expressed GFP (**Figure 1B**). In ear skin single-cell suspensions, c-kit^+^ CD45^+^ CD11b^-^ CD3^-^ MCs percentages and numbers were comparable to *ROSA26* ^LSL-GFP^ controls, and more than 95% of MCs from M-GFP mice were GFP^+^ (**Figures 1C, D**). Frozen sections from shaved back skin of M-GFP mice confirmed the presence of scarce GFP^+^ cells located in the dermis close to hair follicles, consistent with MCs location description (**Figure 1E**) (2).

To assess the labeling of mucosal MCs in M-GFP mice, we analyzed the expression of GFP in c-kit^+^ CD45^+^ MCs from the epithelial layer and lamina propria of the small intestine. Notably, mucosal MCs from M-GFP mice were GFP negative without any significant changes in percentages when compared with controls (**Figures 1F–I**). Thus, we conclude that *Mcpt5* ^Cre^ is active specifically in connective tissue MCs but not mucosal MCs, making it a reliable model to selectively target connective tissue MCs.

### Peritoneal MCs from Heparin deficient mice are reduced in numbers and lack granules

**Figure 2:**
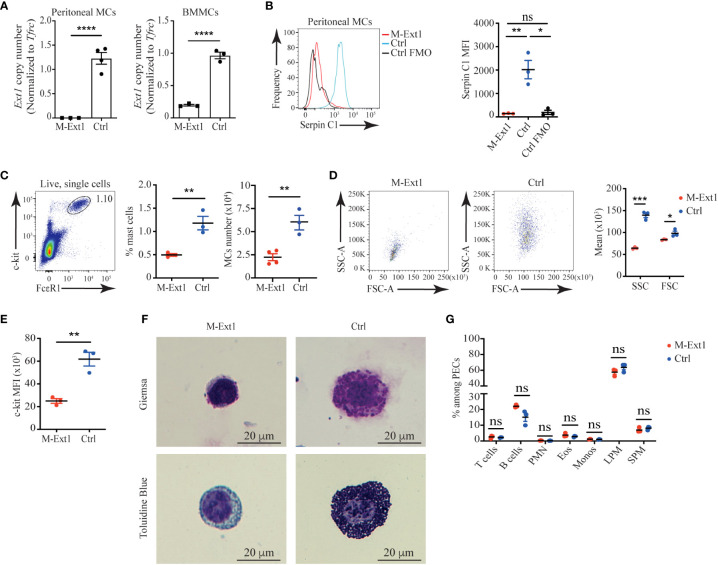
Peritoneal MCs in 6-weeks old Heparin deficient mice are reduced in numbers and lack granules. **(A)** *Ext1* gene copy number analyzed by qPCR from sorted peritoneal MCs and bone marrow-derived mast cells (BMMCs) from M-Ext1 and control mice. **(B)** Representative histograms and statistics of mean fluorescent intensity (MFI) of Serpin C1 binding to fixed and permeabilized peritoneal MCs. Staining of control cells with the omission of the biotinylated SerpinC1 was used as fluorescence minus one (FOM) control. **(C)** Representative gating strategy, percentages and numbers of peritoneal MCs in M-Ext1, and control mice. **(D)** Representative flow cytometry plots and geometric means of forward scatter (FSC) representing size and side scatter (SSC) representing granularity of peritoneal MCs in M-Ext1 and control mice. **(E)** Mean fluorescent intensity of c-kit expression in peritoneal MCs in M-Ext1 and control mice. **(F)** Representative Giemsa and Toluidine Blue staining of c-kit-magnetic beads enriched peritoneal cavity MCs from M-Ext1 and control mice. **(G)** Percentages of different cell populations isolated from the peritoneal cavity determined by flow cytometry; PMN – polymorphonuclear granulocytes, Eos – eosinophils, Monos – monocytes, LPM – large peritoneal macrophages, SPM – small peritoneal macrophages. Results are represented as mean ± SEM. Each symbol in the graphs is an individual mouse. Flow cytometry plots in **(B–E**, **G)** are representative of 3 individual experiments. Images in **(F)** are representative of 3 individual mice of each genotype. Statistical significance was determined by unpaired Student’s t-test. Values of p<0.05 were considered statistically significant, *p < 0.05, **p < 0.01, ***p < 0.001, ****p < 0.0001, ns, not significant.

To eliminate Heparan sulfate and Heparin from connective tissue MCs, we crossed *Mcpt5* ^Cre^ mice with *Ext1* ^f/f^ mice (18). *Ext1* encodes the glycosyltransferase Exostosin 1, which is essential for the synthesis of both HS and Heparin (23). First, we tested the deletion efficiency of *Ext1* in MCs of *Mcpt5* ^Cre^ X *Ext1* ^f/f^ (M-Ext1) mice. *Ext1* ^f/f^ mice were used as controls throughout the study. We could not detect the *Ext1* gene in sorted peritoneal MCs from M-Ext1 mice. However, *Ext1* deletion was incomplete in BMMCs of the same mice (**Figure 2A**). Around 20% of BMMCs had an intact *Ext1* allele (**Figure 2A**). Hence, we conclude that *Ext1* is efficiently deleted only in primary connective tissue MCs but not in ex vivo differentiated BMMCs.

To ensure that Heparin is absent from M-Ext1 MCs, we permeabilized and stained peritoneal exudate cells with biotinylated Serpin C1, which specifically interacts with Heparin (24). While we could clearly detect Serpin C1 binding to control peritoneal MCs, M-Ext1 MCs did not stain with this reagent confirming that *Ext1* deletion results in the absence of Heparin (**Figure 2B**) and validating that M-Ext1 MCs lack Heparin.

Then, we investigated the changes in peritoneal MCs in the absence of Heparin. Peritoneal MCs from 6-weeks-old M-Ext1 mice were significantly decreased in numbers and percentages (**Figure 2C**). Furthermore, our flow cytometry data revealed that Heparin-deficient MCs were smaller and less granular than controls (**Figure 2D**) and had reduced c-kit expression (**Figure 2E**). Giemsa and Toluidine blue staining of c-kit-microbeads enriched peritoneal cells confirmed the great reduction in the granule content in MCs from M-Ext1 mice (**Figure 2F**). Nevertheless, the defects in the peritoneal cavity of M-Ext1 mice were restricted to MCs, as all other cell types appeared in normal proportions (**Figure 2G**).

To find out whether the observed changes in peritoneal MCs were permanent, we analyzed 14-week-old mice and found similar changes in numbers, percentages, granularity, c-kit expression, and Heparin content in M-Ext1 MCs (**Supplementary Figures 1A–E**). Thus, these data suggest that deletion of *Ext1* in peritoneal MCs results in reduced numbers of these cells, defects in their granular content, and lower c-kit expression.

### Reduced numbers and granularity of skin MCs from older M-Ext1 mice

**Figure 3:**
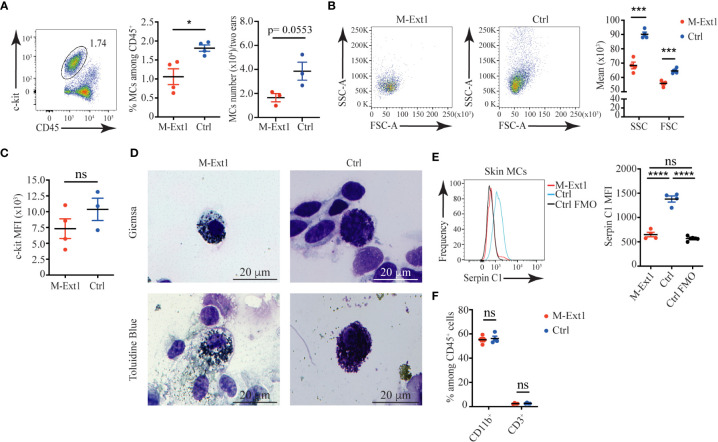
Reduced numbers and granularity in 14-weeks old M-Ext1 skin MCs. **(A)** Representative gating strategy, percentages, and numbers of skin MCs in M-Ext1 and control mice. **(B)** Representative dot plots and graphs of geometric means of size (FSC) and granularity (SSC) of skin MCs. **(C)** Mean fluorescent intensity of c-kit expression in skin MCs in M-Ext1 and control mice. **(D)** Representative Giemsa and Toluidine Blue staining of c-kit-magnetic beads enriched skin MCs from M-Ext1 and control mice. **(E)** Representative histograms and statistics of mean fluorescent intensity (MFI) of Serpin C1 binding to fixed and permeabilized skin MCs from M-Ext1 and control mice. **(F)** Percentages of CD11b^+^ and CD3^+^ cells in the skin from M-Ext1 and control mice. Results are represented as mean ± SEM. Each symbol in the graphs is an individual mouse. Flow cytometry plots in **(A–C**, **E)** are representative of 4 individual experiments. Images in **(D)** are representative of 3 individual mice of each genotype. Statistical significance was determined by unpaired Student’s t-test. Values of p<0.05 were considered statistically significant, *p < 0.05, ***p < 0.001, ****p < 0.0001, ns, not significant.

To find out if other connective tissue MCs in M-Ext1 mice have defects, we characterized skin MCs. Surprisingly, MCs from 6-weeks-old M-Ext1 mice showed no difference in numbers, percentages, size, granularity, and c-kit surface expression compared to control mice (**Supplementary Figures 2A–C**). Giemsa and Toluidine Blue staining showed MCs with large numbers of granules in their cytoplasm (**Supplementary Figure 2D**), despite the efficient deletion of *Ext1* evidenced by the absence of Serpin C1 binding to skin MCs from young M-Ext1 mice (**Supplementary Figure 2E**). In contrast, MCs from 14-weeks-old M-Ext1 mice were reduced in proportions and numbers (**Figure 3A**) and had decreased granularity and size (**Figures 3B, D**), although they did not express statistically significantly lower levels of c-kit on their surface (**Figure 3C**). Consistently, Heparin could not be detected with Serpin C1 staining inside the cells, suggesting efficient deletion of *Ext1* (**Figure 3E**). The percentages of CD11b^+^ myeloid cells and CD3^+^ T cells in the skin were not changed in M-Ext1 mice. Hence the defects in the skin are limited to the MC pool (**Figure 3F** and **Supplementary Figure 2F**).

### Heparin plays no major role in recruiting inflammatory cells during sterile peritonitis and the pathogen clearance during bacterial peritonitis

**Figure 4:**
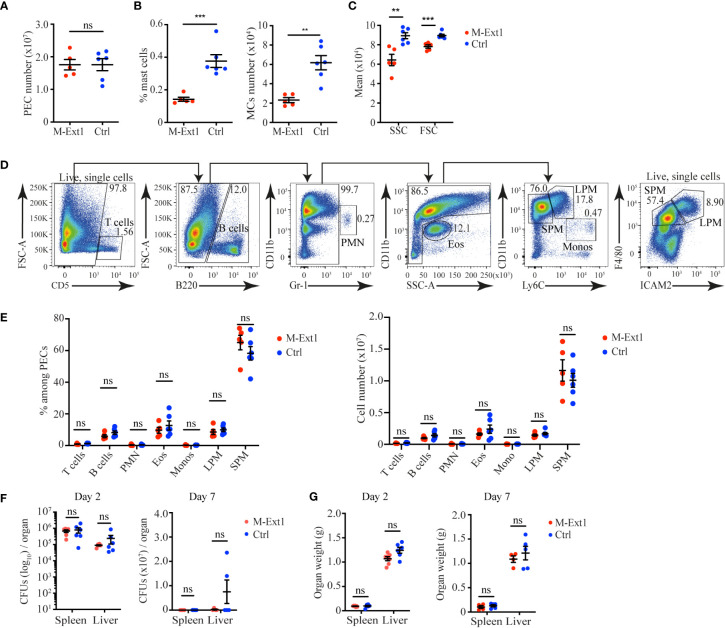
Unimpaired recruitment of inflammatory cells in M-Ext1 mice during sterile peritonitis and pathogen clearance during bacterial peritonitis in 6-weeks old mice. **(A)** The total number of peritoneal exudate cells isolated from the peritoneal cavity of M-Ext1 and control mice at day 4 post intraperitoneal injection with 4% Thioglycolate. **(B)** MCs percentages and numbers among peritoneal cells at day 4 after Thioglycolate injection in M-Ext1 and control mice. **(C)** Geometric means of size (FSC) and granularity (SSC) of peritoneal cavity MCs from M-Ext1 and control mice injected intraperitoneally with 4% Thioglycolate 4 days in advance. **(D)** Gating strategy for identifying different populations within the peritoneal exudate cells. **(E)** Percentages and numbers of different cell populations isolated from the peritoneal cavity of M-Ext1 and control mice injected intraperitoneally with 4% Thioglycolate 4 days in advance. **(F)** Bacterial burden in spleens and livers from M-Ext1 and control mice infected intraperitoneally with *L. monocytogenes* determined at day 2 and day 7 post-infection. **(G)** Weights of spleens and livers from *L. monocytogenes* infected M-Ext1 and control mice determined at day 2 and day 7 post-infection. Results are represented as mean ± SEM. Each symbol in the graphs is an individual mouse. Data in **(A–C**, **E)** are from 5-6 mice per group. Flow cytometry plots in **(D)** are representative of 6 individual experiments. Data in **(F, G)** are from 5-6 mice per group. Statistical significance was determined by unpaired Student’s t-test, except for **(F)**, in which non-parametric Mann-Whitney test was used. Values of p<0.05 were considered statistically significant, **p<0.01, ***p<0.001, ns, not significant.

To determine the importance of Heparin in physiological settings, we decided to use two mouse models of peritonitis, in which the roles of MCs have been characterized previously. Recruitment of inflammatory cells during sterile peritonitis has been shown to depend on MCs (25). To test the role of Heparin in the process, we injected 6 weeks-old M-Ext1 mice and controls intraperitoneally with 4% Thioglycolate and 4 days later analyzed the peritoneal exudate cells (PECs) by flow cytometry. The total numbers of PECs were indistinguishable between M-Ext1 and control mice (**Figure 4A**). MCs percentages, numbers, size, and granularity were still reduced in the Heparin-deficient mice compared with controls (**Figures 4B, C**). However, there were no statistically significant changes in the percentages and numbers of any other cell type (**Figures 4D, E**), suggesting that Heparin and MCs granules are dispensable for the recruitment of inflammatory cells at d. 4 of Thioglycolate induced peritonitis. We observed a similar MCs phenotype in 14-weeks-old Heparin deficient mice (**Supplementary Figures 3A–C**) and did not find any differences in the proportions and numbers of any PECs populations (**Supplementary Figures 3D, E**). Therefore, we conclude Heparin is not required for inflammatory cell recruitment during sterile peritonitis.

To test the role of Heparin in bacterial peritonitis, we used intraperitoneal *L. monocytogenes* infection as MCs depletion enhances susceptibility to *L. monocytogenes* peritonitis (26). We infected 6 weeks-old M-Ext1 and control mice intraperitoneally with *L. monocytogenes* and determined the bacterial burden at days 2 and 7 post-infection. We found comparable bacterial burden and organ weight in Heparin-deficient and control mice (**Figures 4F, G**). Hence, we conclude that Heparin is not required for *L. monocytogenes* infection clearance in the peritoneal cavity. Thus, in both peritonitis models that we used, the absence of Heparin was dispensable for the recruitment of inflammatory cells and the clearance of the pathogen.

### Heparin enhances the spread of skin infection

**Figure 5:**
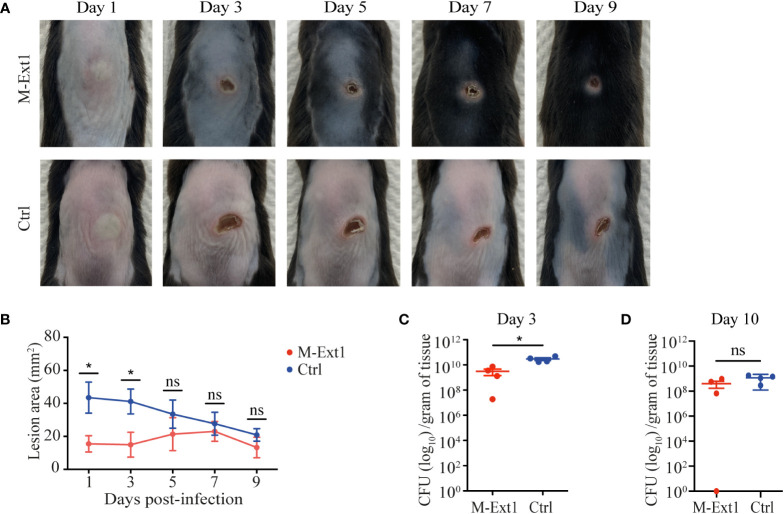
Heparin enhances the spread of skin infection. **(A)** Representative images of skin lesions from M-Ext1 and control mice at the indicated time points post i.d. infection with 10^7^ *S. aureus* strain JE2. **(B)** Comparison of the areas of skin lesions in infected M-Ext1 and control mice at indicated times. **(C)** Bacterial burden in homogenized skin tissue surrounding the lesions determined at day 3 post-infection in infected M-Ext1 and control mice. **(D)** Bacterial burden in homogenized skin tissue surrounding the lesions at day 10 post-infection in M-Ext1 and control mice. Results are represented as mean ± SEM from 4 mice of each genotype. Each symbol in the graphs is an individual mouse. Images in **(A)** are representative of 4 individual mice. Statistical significance in **(B)** was determined by unpaired Student’s t-test comparing M-Ext1 to control mice at each time point. Statistical significance in **(C, D)** was determined by two-tailed Mann-Whitney test. Values of p<0.05 were considered statistically significant, *p < 0.05, ns, not significant.

To test the importance of skin MC Heparin in combatting bacterial infection, we used necrotizing dermatitis caused by *S. aureus*. The absence of MCs promotes the spread of skin lesions in this model (27). We infected M-Ext1 and control mice intradermally with *S. aureus* strain JE2 and measured the lesion area every 2 days post-infection. Surprisingly, the skin lesions on M-Ext1 mice were smaller than control mice (**Figures 5A, B**). The difference was the greatest at early time points (day 1-3) and disappeared after day 5. Corresponding to the skin lesions, the bacterial burden in the tissue was significantly lower in M-Ext1 mice on day 3 (**Figure 5C**), while there was no difference on day 10 (**Figure 5D**). Thus, the absence of Heparin facilitates bacterial killing and limits the spread of necrotizing dermatitis caused by *S. aureus* at early time points.

### Heparin deficiency augments skin inflammation upon irritant challenge

**Figure 6:**
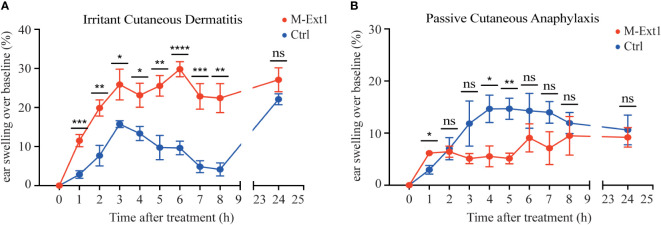
Heparin deficiency augments skin inflammation upon irritant challenge but not in PCA model in 14-weeks old mice. **(A)** The change of ear thickness over baseline between M-Ext1 and control mice at the indicated time points after 0.5% DNFB application. **(B)** The change of ear thickness over baseline between M-Ext1 and control mice after i.v. injection of chicken ovalbumin (Ova) following prior sensitization with mouse anti-Ova IgE of M-Ext1 and controls mice. Results are represented as mean ± SEM from 3-6 mice of each genotype. Each symbol in the graphs is an individual mouse. Statistical significance was determined by unpaired Student’s t-test. In **(A, B)**, the comparison between M-Ext1 and control mice was done for each time point. Values of p<0.05 were considered statistically significant, *p < 0.05, **p < 0.01, ***p < 0.001, ****p < 0.0001, ns, not significant.

The smaller lesion size of *S. aureus* necrotizing dermatitis in M-Ext1 mice was unexpected and prompted us to further explore the importance of Heparin in skin MCs. First, we turned to an inflammatory model that depends exclusively on MCs – the irritant dermatitis. Application of the irritant DNFB to the skin on mouse ears without prior sensitization leads to ear swelling within hours, which is abolished in MC-deficient mice (5). Indeed, DNFB painting induced ear swelling that peaked at 3 h in control 14-weeks-old mice (**Figure 6A**). However, the ear swelling was much greater and occurred faster in M-Ext1 mice, suggesting that the absence of Heparin enhances inflammation. This phenomenon occurred only in older (14-weeks-old) but not in younger mice (6-weeks-old) M-Ext1 mice, in which the ear swelling was identical to controls (**Supplementary Figure 4A**). This result is consistent with the absence of skin MCs defects in younger mice. Another model that relies on skin MCs for inducing inflammation is PCA (28). Injection of a model antigen (chicken ovalbumin – Ova) caused ear swelling in mice pre-sensitized with anti-Ova IgE (**Figure 6B**). In contrast to the irritant dermatitis, the response to IgE crosslinking was significantly decreased at several time points in 14-weeks old Heparin-deficient mice. No difference from controls was observed in younger, 6 weeks old mice in PCA (**Supplementary Figure 4B**). Taken together, our results indicate that M-Ext1 mice have exaggerated skin response to irritants but not FcεR1 ligation.

### Heparin-deficient skin MCs contain less Histamine and TNFα but have enhanced expression of *Mrgprb2*

**Figure 7:**
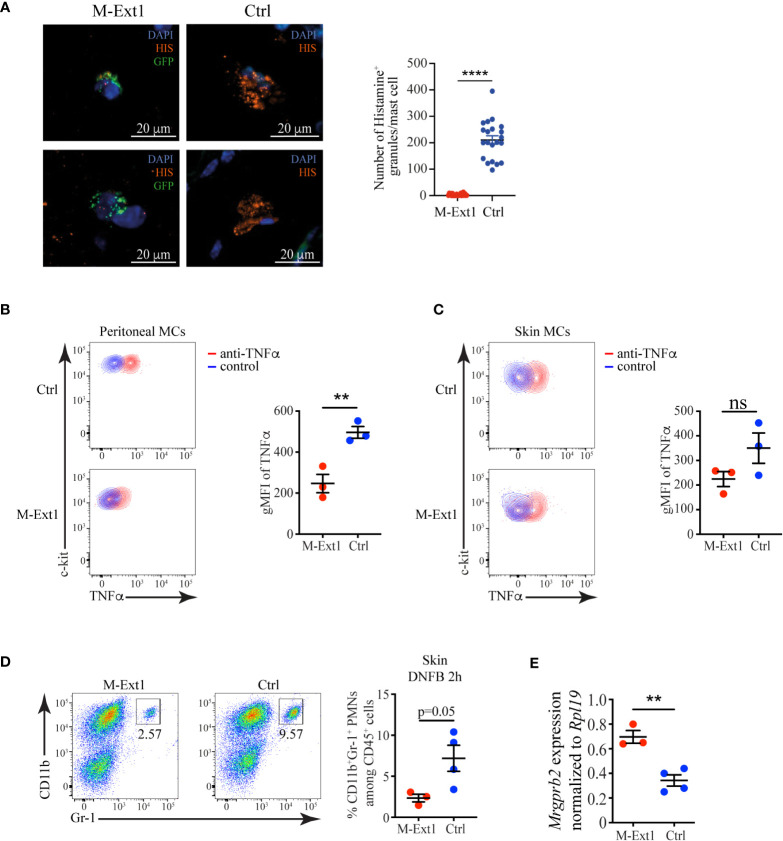
Reduced levels of Histamine and TNFα, but an enhanced expression of *Mrgprb2* in Heparin-deficient mast cells. **(A)** Immunofluorescent staining for Histamine of skin sections from M-Ext1 crossed to *ROSA26^LSL-GFP^* and control (*Ext1* ^f/f^) mice and quantification of the number of Histamine^+^ granules in skin mast cells from both genotypes. Histamine^+^ granules were counted in 3-4 fields of view from sections from three different mice per genotype. **(B)** Flow cytometry plots showing TNFα expression in peritoneal mast cells and comparison of the geometric mean fluorescent intensity (gMFI) between M-Ext1 and control peritoneal mast cells. **(C)** Flow cytometry plots showing TNFα expression in skin mast cells and comparison of the geometric mean fluorescent intensity (gMFI) between M-Ext1 and control skin mast cells. **(D)** Flow cytometry plots showing the abundance of CD11b^+^Gr1^+^ monocytes 2 h after DNFB application on the ears of M-Ext1 and control mice. The numbers inside the flow cytometry plots are the percentages of neutrophils among CD45^+^ hematopoietic cells. **(E)** *Mrgprb2* mRNA expression determined by qPCR from sorted skin MCs from M-Ext1 and control mice. Results are represented as mean ± SEM from 3-4 mice of each genotype. Each symbol in the graphs is an individual mouse. Statistical significance was determined by unpaired Student’s t-test. Values of p<0.05 were considered statistically significant, **p < 0.01, ****p < 0.0001, ns, not significant.

We assessed several critical inflammatory mediators stored in mast cell granules to understand the mechanism behind the enhanced ear-swelling upon DNFB application in M-Ext1 mice. Histamine is one of the most important biogenic amines and regulates vascular permeability, smooth muscle contraction, and many other biological processes (29). We used immunofluorescent staining of skin sections from M-Ext1 and control mice to reveal the abundance of Histamine in skin mast cells. To identify the mast cells in mutant mice, we crossed them to *ROSA26* ^LSL-GFP^ so that connective tissue mast cells would express GFP. Each control mast cell contained numerous granules staining with anti-Histamine antibody (**Figure 7A**). However, Histamine was barely detectable in mast cells lacking Heparin, and very few Histamine-positive granules could be detected in the skin of these mice. Another essential inflammatory mediator stored in mast cell granules is the cytokine TNFα. It is critically important for mast cell-mediated neutrophil recruitment (30). TNFα was detectable in peritoneal and skin mast cells (**Figures 7B, C**), but it was expressed at significantly lower levels in Heparin-deficient mast cells compared to controls. Moreover, neutrophil recruitment 2 hours after DNFB application to the skin was reduced in M-Ext1 mice (**Figure 7D**), confirming the indispensable role of TNFα in neutrophil recruitment. Thus, both Histamine and TNFα were reduced in Heparin-deficient mast cells and cannot explain the increased ear swelling following skin irritants application and the smaller lesion size in *S. aureus* necrotizing dermatitis in M-Ext1 mice.

The differential responsiveness of M-Ext1 mice to irritant dermatitis caused by DNFB and IgE-mediated PCA prompted us to investigate the expression of *Mrgprb2*. *Mrgprb2* is a receptor for basic secretagogues such as several irritants, peptides, and toxins, including *S. aureus* δ-toxin, that can activate MCs independently of FcεR1 (31, 32). We sorted skin MCs from 14-week-old mice and evaluated the expression of *Mrgprb2* by qPCR. We found that *Mrgprb2* expression was significantly up-regulated in M-Ext1 MCs compared to controls (**Figure 7E**), potentially explaining the enhanced sensitivity of M-Ext1 MCs to irritants such as DNFB.

## Discussion

Here, we show that Heparin deficiency has a profound effect on connective tissue MCs. The absence of Heparin resulted in reduced granule content and a lower number of MCs in the peritoneal cavity and the skin. MC Heparin was not required to recruit inflammatory cells during sterile peritonitis nor for pathogen clearance during bacterial peritonitis. However, necrotizing dermatitis lesions caused by *S. aureus* were smaller and had a lower bacterial burden in Heparin-deficient mice, and the mice exhibited exaggerated irritant dermatitis. We ruled out increased production and storage of Histamine and TNFα as reasons for the altered functions of the Heparin-deficient mast cells. However, these cells featured overexpression of the irritant receptor *Mrgprb2*, which could potentially explain the enhanced responsiveness of M-Ext1 mice to DNFB and *S. aureus* producing the *MrgprB2* ligand δ-toxin (32).

Just as described before in the *Ndst2* ^-/-^ strains (11, 10), the absence of Heparin in M-Ext1 mice diminished the number of MCs and their granularity, confirming the requirement for Heparin in packaging the granule mediators. An important difference between the two mouse strains is that *Ndst2* ^-/-^ mice lack only sulfation but not the HS backbone of Heparin. It is possible that the unsulfated HS chains on Serglycin maintain some of Heparin’s functions. Nevertheless, the similarity in the phenotype between the two mouse strains argues that Heparin sulfation is essential for the packaging of MC granules.

Although first demonstrated more than 20 years ago, the requirement of Heparin for MC survival has not been completely explained. The leading theory is that the enzymatically active proteases need to be restrained by Heparin to prevent proteolytic damage. However, ~50% of MCs survive without any Heparin indicating adaptation of these cells most likely through down-regulation of protease expression as has been demonstrated for Mcpt4, Mcpt5, Mcpt6, and Cpa (10‐12). An additional factor for the lower number of MCs could be the lower expression of c-kit, the essential growth factor for MCs that has also been reported by Abrink et al. (12). It was surprising that peritoneal MCs were reduced in both young and older mice, but skin MCs were found in normal numbers in young (6 weeks) but not in older (14 weeks) mice. The reason for this discrepancy might lie in the developmentally programmed increase in Heparin content (20) and the existence of a threshold above which Heparin-unbuffered proteases might become toxic. Alternatively, potential differences in the reliance on Heparin and the maintenance of MCs from different origins (early erythro-myeloid progenitors vs. late erythro-myeloid progenitors vs. adult hematopoietic stem cells) could also be at play (33).

Surprisingly, despite their reduced numbers of MCs and lack of granules, Heparin-deficient mice responded stronger to certain stimuli. In irritant dermatitis induced by DNFB, the ear swelling was greater in M-Ext1 mice than in controls. However, the enhanced response could not be attributed to higher production of Histamine or TNFα-mediated neutrophil recruitment, as both of these mediators were lower in Heparin-deficient mast cells. One possibility is that increased Tryptase release from these cells could account for the increased swelling as Tryptase facilitates edema formation (34). Moreover, *Ndst2* ^-/-^ mice seem to have higher mRNA levels of *Mcpt7*, which is a Tryptase (11). Unfortunately, the Tryptase activity in sorted primary mast cells was below the detection threshold.

Exaggerated MCs response has been previously observed in mice deficient for Langerhans cells, specialized antigen-presenting cells in the epidermis (27). In this study, skin MCs were found to express higher levels of the receptor *Mrgprb2*, which is responsible for FcεR1-independent MCs degranulation following a variety of stimuli, including antibiotics, DNCB, croton oil, house dust mite, Substance P, and so on (27, 31) (35, 36). Thus, upregulation of *Mrgprb2* could account for the increased ear swelling in response to DNFB and the reduced lesion size in the *S. aureus* necrotizing dermatitis model in M-Ext1 mice. Zhang et al. showed that the absence of Langerhans cells caused a reduction in *MrgprD*-expressing skin neurons that normally suppress MC via glutamate release in the skin (27). Whether this is the case in M-Ext1 mice remains to be determined.

Another explanation for the enhanced killing of bacteria in M-Ext1 mice could lie in the ability of Heparin to dismantle neutrophil extracellular traps (NETs) (37). The highly negatively charged Heparin can interact with and neutralize the positive charge of histones – major components of NETs (38). Thus, in the absence of Heparin in M-Ext1 mice, there will be greater NETs formation, a higher level of inflammation, and enhanced bacterial clearance. However, this phenomenon only appears influential in the first 3-5 days.

The enhanced ear swelling following DNFB application in M-Ext1 mice was in contrast to the decreased response of these mice in the PCA model. This outcome could result from the reduced number of MCs combined with a normal reaction to FcεR1 ligation. The activation of MCs by IgE crosslinking in Langerhans cells-deficient mice was also normal despite the heightened sensitivity to *MrgprB2* ligands (27). Moreover, PCA severity was slightly reduced but still present in *Ndst2* ^-/-^ mice (11). Thus, our findings feed into a model of at least two independent activation modules in MCs – FcεR1-induced and MrgprB2-induced.

Despite the reduction in peritoneal cavity MCs, we could not find a defect in the immune cell recruitment in sterile peritonitis or the pathogen clearance in bacterial peritonitis. These data are consistent with the normal recruitment of neutrophils after IgE injection in *Ndst2* ^-/-^ mice (10). The role of MCs in protection against bacterial infection in the peritoneum is controversial. Early studies found a critical protective role for MCs (39). However, more recent work has pointed to detrimental functions of these cells, for example, inhibition of macrophage phagocytosis (40). Additional stimuli and time points need to be tested to conclusively determine the role of Heparin in mast cells for inflammatory cell recruitment.

In summary, our results clarify some of the physiological functions of Heparin and will, undoubtedly, open new avenues of research into the roles of MCs and their granules in health and disease.

## Data availability statement

The raw data supporting the conclusions of this article will be made available by the authors, without undue reservation.

## Ethics statement

The animal study was reviewed and approved by The Institutional Animal Care and Use Committee of National Yang Ming Chiao Tung University.

## Author contributions

SAH-H designed and performed all the experiments, interpreted data, and wrote the manuscript; H-PH biotinylated and tested Serpin C1 as a tool to detect Heparin; Y-HT provided guidance in bacterial infection models; C-YK provided *S. aureus* for necrotizing dermatitis model; YY provided the *Ext1* ^f/f^ mice; AR provided *Mcpt5* ^Cre^ mice; C-LH conceptualized research and designed experiments; ID conceptualized research, designed experiments, interpreted data, and wrote the manuscript. All authors contributed to the article and approved the submitted version.

## Funding

This work was supported by the Ministry of Science and Technology (MOST) grants 106-2320-B-010- 026-MY3, 107-2320-B-010-016-MY3, and 110-2320-B-A49A-521 – to ILD and grants from the Yen Tjing Ling Medical Foundation, CI-107-6, CI-108-5, and CI-111-6 to ID.

## Acknowledgments

We would like to thank Dr. Fang Liao (Academia Sinica, Taiwan) for 24G2 hybridoma; Dr. Jie-Rong Huang (NYCU, Taiwan) for help with protein purification and biotinylation; Dr. Rebecca Gentek (Aix-Marseille University, France) for sharing the Avidin-AF488 staining protocol; Dr. Shih-Lien Wang (Tzu-Chi University, Taiwan) for help with *Listeria monocytogenes* experiments. The following reagent was provided by the Network on Antimicrobial Resistance in *Staphylococcus aureus* (NARSA) for distribution by BEI Resources, NIAID, NIH: *Staphylococcus aureus subsp. aureus*, Strain JE2, NR-46543.

## Conflict of interest

The authors declare that the research was conducted in the absence of any commercial or financial relationships that could be construed as a potential conflict of interest.

## Publisher’s note

All claims expressed in this article are solely those of the authors and do not necessarily represent those of their affiliated organizations, or those of the publisher, the editors and the reviewers. Any product that may be evaluated in this article, or claim that may be made by its manufacturer, is not guaranteed or endorsed by the publisher.

## Abbreviations

MC, mast cell; HS, Heparan sulfate; *Ndst2*, N-deacetylase/N-sulfotransferase 2; M-GFP, *Mcpt5* ^Cre^ X *ROSA26* ^LSL-GFP^; M-Ext1, *Mcpt5* ^Cre^ X *Ext1* ^f/f^; BMMC, bone marrow-derived mast cell; NETs, neutrophil extracellular traps; PCA, passive cutaneous anaphylaxis.
