# Supplementary figures and images for: Heparin is required for the formation of granules in connective tissue mast cells
Source: Front Immunol. 2022 Nov 9;13:1000405. doi: 10.3389/fimmu.2022.1000405 (PMC9682282; doi:10.3389/fimmu.2022.1000405)

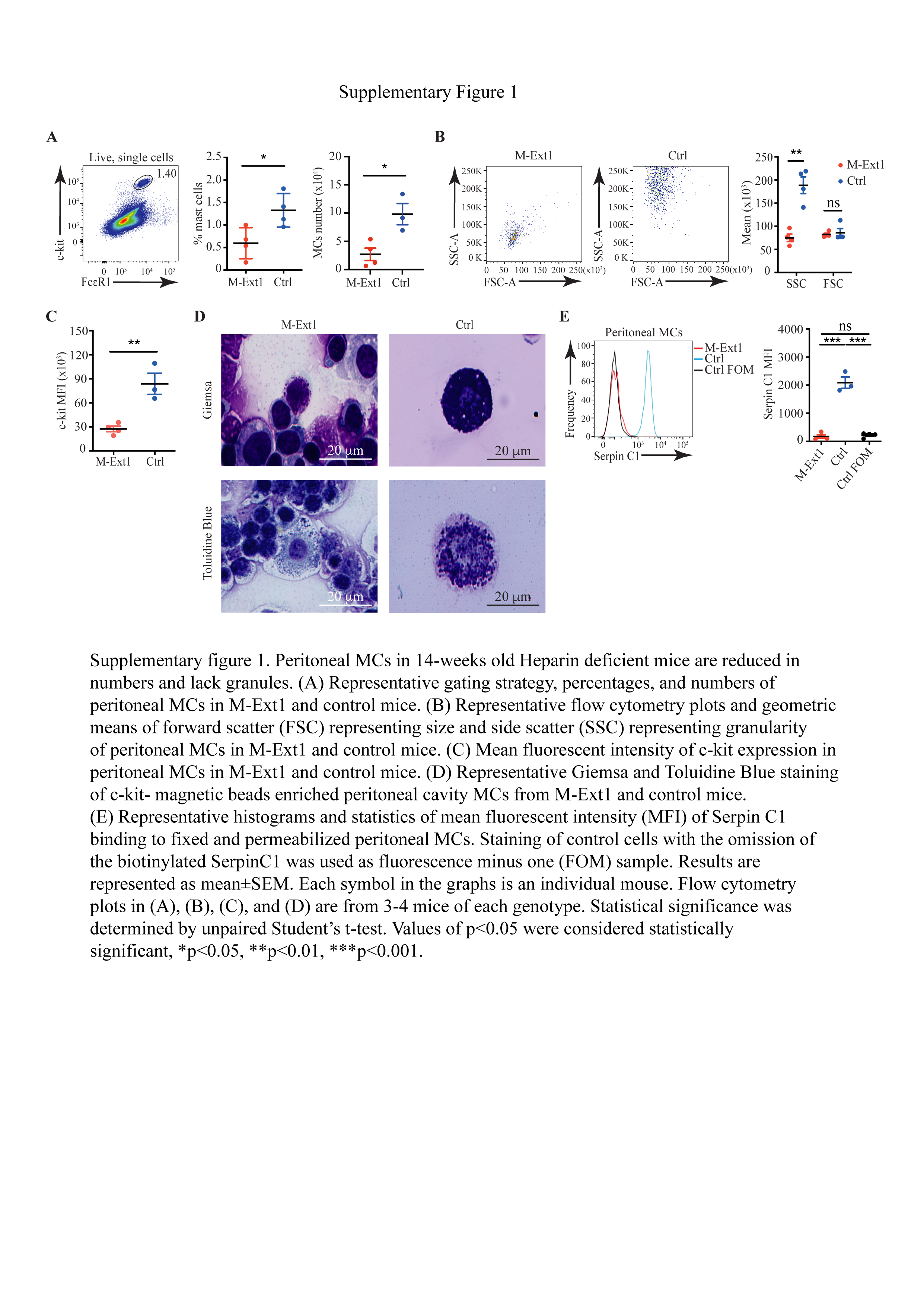

Supplement: Supplementary file 1 [file Image_1.tif]

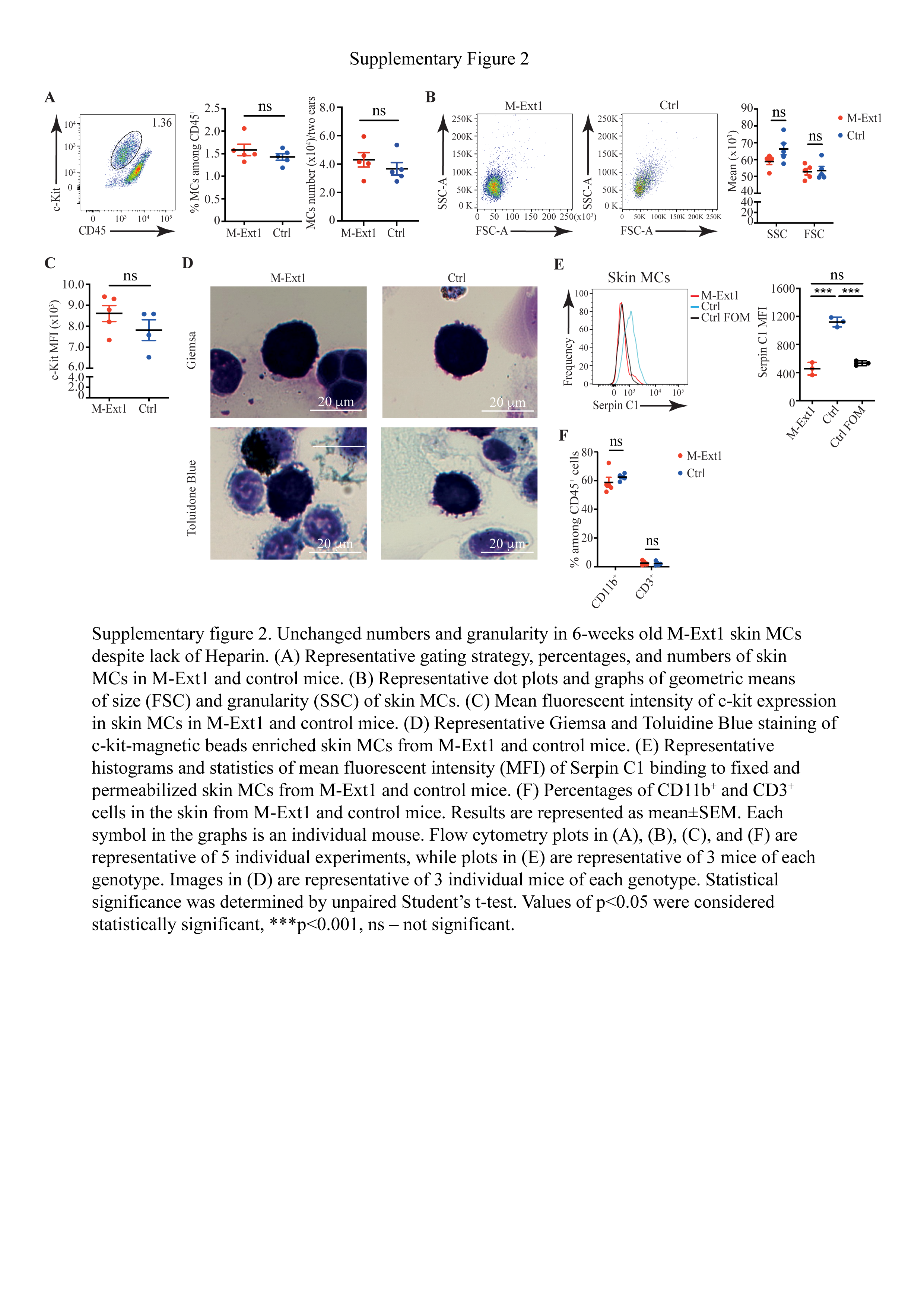

Supplement: Supplementary file 2 [file Image_2.tif]

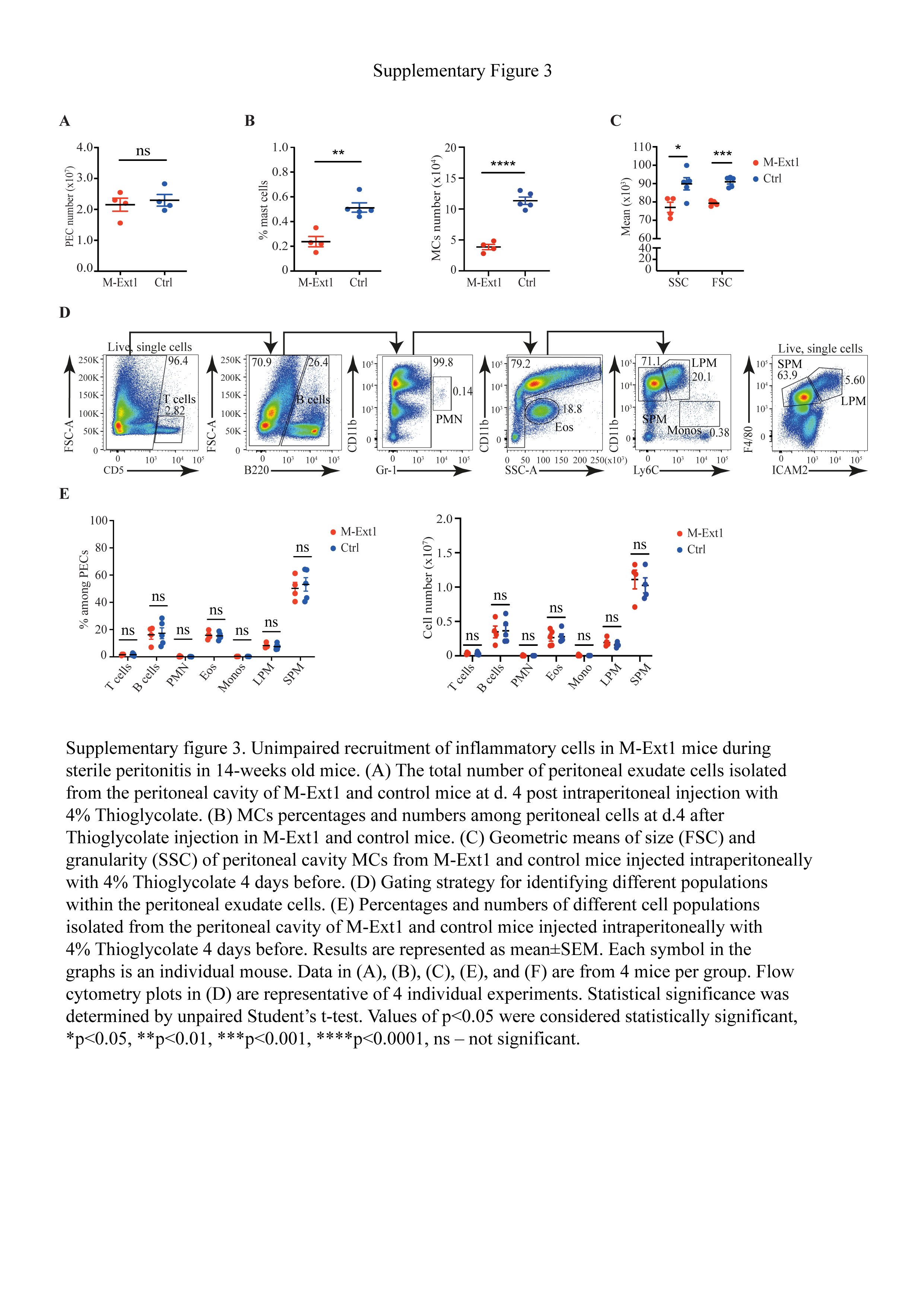

Supplement: Supplementary file 3 [file Image_3.tif]

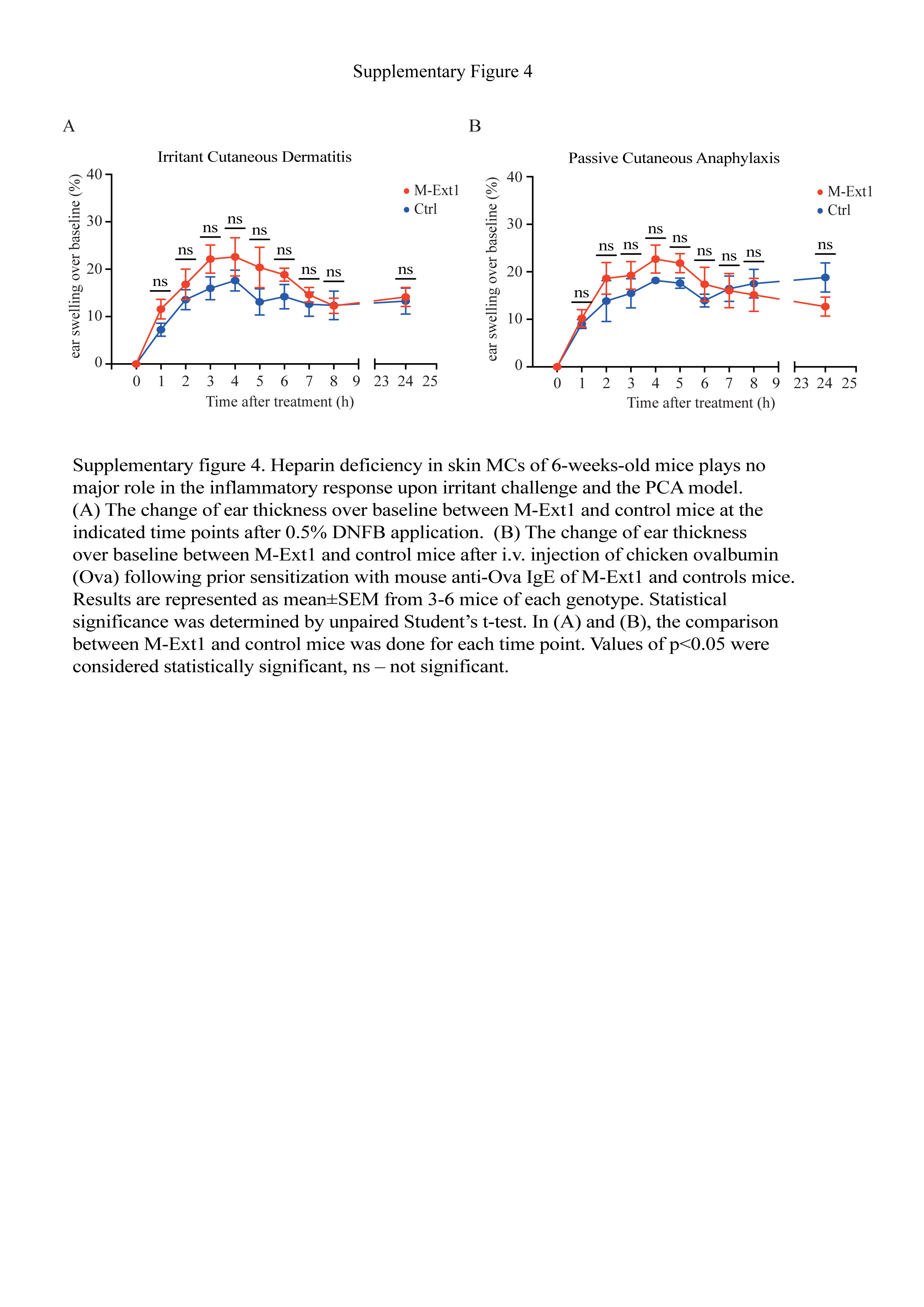

Supplement: Supplementary file 4 [file Image_4.tif]
